# Investigating use of diagnostic codes for post-COVID- 19 condition in Ontario health administrative data

**DOI:** 10.1186/s12913-025-12751-4

**Published:** 2025-05-14

**Authors:** Joseph S. Munn, Clare L. Atzema, Peter C. Austin, Stacey J. Butler, Lee Fidler, Xuesong Wang, Andrea S. Gershon

**Affiliations:** 1 Sunnybrook Research Institute, Sunnybrook Health Sciences Centre, G1 06 - 2075 Bayview Avenue, Toronto, ON M4N 3M5 Canada; 2 ICES, Toronto, ON Canada; 3 Department of Medicine, University of Toronto, Toronto, ON Canada; 4 Institute of Health Policy, Management and Evaluation, University of Toronto, Toronto, ON Canada; 5 Institute of Medical Science, University of Toronto, Toronto, ON Canada; 6 Division of Respirology, Sunnybrook Health Sciences Centre, Toronto, Canada; 7 Division of Respirology, University Health Network, Toronto, Canada

**Keywords:** Post-COVID- 19 condition, Diagnostic code evaluation

## Abstract

**Background:**

In January 2023 the Ontario Health Insurance Plan (OHIP) introduced a diagnostic code for post-COVID-19 condition (PCC). We used this code to estimate the incidence rate of PCC, to compare demographic and clinical characteristics of individuals who received a PCC code to those who didn’t, and to investigate healthcare utilization of individuals who received a PCC code.

**Methods:**

We conducted a retrospective cohort study using health administrative data from Ontario, Canada (population approximately 15 million). Individuals who had received a PCC diagnostic code between January 2023 and January 2024 were identified using OHIP, a physician billing database. For the entire population of Ontario, crude incidence rates of PCC were computed and patient characteristics (including age, sex, geographic location, comorbidities, and marginalization index) were collected and compared between individuals who had received a PCC code and those who hadn’t using logistic regression models. Healthcare utilization rates for people who received a PCC code were compared pre-pandemic (January 1st, 2017 to March 31st, 2020), pre-PCC (April 1st, 2020 to 24-weeks pre-PCC diagnostic code), and post-PCC (24-weeks pre-PCC diagnostic code to study end).

**Results:**

A PCC code was received by 7,343 individuals. Median age was 62, and 60% were female. When compared to the entire population of Ontario, female sex, older adults, Northern Ontario residents, and comorbid individuals had greater odds of receiving a PCC code. People who were visible minorities, immigrants, and had less access to material resources had lower odds of receiving a PCC code. Healthcare utilization rates, pre-pandemic, pre-PCC, and post-PCC were 14.59 (CI 13.63–15.61), 27.43 (CI 25.01–30.27), and 100.61 (CI 93.39-107.73) encounters per person-year respectively.

**Conclusion:**

The number of cases captured was lower than what Health Canada estimates would indicate and it is likely that the code is underrepresenting PCC in Ontario. The substantial increases in healthcare utilization suggests the code is capturing severe PCC cases. The characteristics of the cohort were similar to what has been described in peer-reviewed literature, suggesting that the patients in this cohort have PCC. This code could offer a promising way to study a large diverse population of people with PCC.

**Supplementary Information:**

The online version contains supplementary material available at 10.1186/s12913-025-12751-4.

## Background

COVID- 19 continues to place a significant burden on patients and healthcare providers. There were 134,091 confirmed COVID-19 cases reported in Ontario, Canada in 2023 alone, with the true number likely to be substantially higher as testing availability was heavily curtailed [1] with publicly available polymerase chain reaction (PCR) testing no longer offered after November 21st, 2021 [1]. In addition to acute cases, post-COVID-19 condition (PCC), also known as long-COVID, remains prevalent amongst Canadians [2]. PCC is defined by the World Health Organization as experiencing continued or new symptoms three months following an acute infection which cannot be explained by any other cause, with symptoms lasting for at least two months [3]. The most common symptoms attributed to PCC are shortness of breath, fatigue, cardiovascular irregularities, musculoskeletal symptoms, and cognitive difficulties [4–10]. It is estimated that per infection between 10 and 20% of adults experience long-term COVID-19 symptoms with this number increasing dramatically as an individual’s number of acute COVID-19 infections increases, with estimates of 25.4% and 37.9% for 2 and ≥ 3 acute infections respectively [5, 11, 12]. This suggests that conservatively 13,000–26,000 people in Ontario, Canada will have acquired PCC in 2023. Several risk factors for PCC have been established in the literature, including female sex, older age, number and severity of COVID-19 infections, disabilities, and comorbidities [4, 5].

At present, the PCC literature is limited and the majority of clinical PCC studies have small sample sizes, highly heterogeneous clinical definitions of PCC, and limited follow up time [6, 13]. Population-based studies with large sample sizes that make use of health administrative data have, relied on comparing long-term healthcare utilization rates in individuals with laboratory-confirmed COVID-19 (versus test-negative controls) as they were unable to establish a PCC diagnosis [14–16]. This is problematic as the increase in healthcare utilization cannot necessarily be attributed to PCC in individual patients as it is possible that this increase may be the result of chronic disease or other healthcare utilization. This lack of certainty makes it difficult to identify factors uniquely associated with PCC with a high degree of confidence.

In January 2023, the Ontario Ministry of Health introduced a PCC diagnostic code for the publicly-funded Ontario Health Insurance Plan (OHIP), enabling physicians to specifically code and bill for PCC. This new code represents a potential method for monitoring PCC using health administrative data, allowing for the creation of surveillance protocols and the large scale and long-term study of PCC. The purpose of this study was to investigate the new PCC code to enable researchers to study PCC and perform real-world surveillance using the code. The primary objectives were to describe the demographics, clinical characteristics, and healthcare utilization patterns of patients who received a PCC code. The secondary objective was to determine which physician specialties used PCC codes.

## Methods

### Study design and setting

We conducted a population-based cohort study between January 1 st 2023 and January 1 st 2024, using health administrative data from all residents of Ontario, the most populous province in Canada (approximately 15 million residents). Ontario has a publicly funded healthcare system where almost all residents of Ontario are insured for medically necessary healthcare by OHIP. Individuals not covered by OHIP include federal prisoners, some indigenous populations living on reservations, and military personnel who are not permanent residents of Ontario.

### Data sources

A total of 35 health administrative and insurance claims databases were used for this study, they are described in detail in the Supplementary material. These databases have been validated for accuracy, are regularly updated, and collectively contain demographic information, health services use (physician claims, ambulatory care, and hospitalization discharge databases), physician characteristics, COVID-19 PCR test data, and COVID-19 vaccination data [17–19] for almost all people in Ontario. All COVID-19 vaccinations that were delivered in Ontario were captured. This includes vaccination date, but not the specific vaccine delivered. PCR test date and result (positive or negative) of all publicly funded COVID-19 PCR tests performed by a healthcare professional in a healthcare facility or testing center in the province of Ontario are collected by the Ontario Ministry of Health.

The databases used for this study were accessed at ICES (formerly the Institute for Clinical Evaluative Sciences). ICES is an independent, non-profit research institute whose legal status under Ontario’s health information privacy law allows it to collect and analyze health care and demographic data, without consent, for health system evaluation and improvement. These datasets were linked using unique encoded identifiers and analyzed at ICES.

### Ethics approval and consent to participate

ICES is a prescribed entity under Ontario’s Personal Health Information Protection Act (PHIPA). A waiver of consent was received for this study as, Sect. 45 of PHIPA authorizes ICES to collect personal health information, without consent, for the purpose of analysis or compiling statistical information with respect to the management of, evaluation or monitoring of, the allocation of resources to or planning for all or part of the health system. Projects that use data collected by ICES under section 45 of PHIPA, and use no other data, are exempt from research ethics board review. The use of the data in this project is authorized under section 45 and approved by ICES’ Privacy and Legal Office.

This study adhered to the national and provincial guidelines of Ontario, Canada as established by PHIPA.

### Study cohort

This study used two cohorts, a patient cohort and a physician cohort.

### Patient cohort

Individuals were included in the patient cohort if they were eligible for OHIP, resided in the province of Ontario, and received at least one OHIP diagnostic code (081) or an International Classification of Diseases Tenth Revision (ICD-10) diagnostic code (U07.4) for PCC between January 1, 2023 and January 1, 2024 [20]. However, the OHIP database was not fully updated in December 2023 at the time of the study and may not contain all patient encounters. It was decided to include December despite this so as to capture as many individuals who had received a PCC code as possible. In Ontario individuals can receive an OHIP code as a result of an outpatient visit (in person or virtual), emergency department (ED) visit, or hospitalization. An individual can only receive an ICD-10 code in the ED, during same day surgery (SDS) or as an inpatient in hospital. The index date for patients in the study was the date that their first PCC code was received.

### Physician cohort

The physician cohort included all physicians who used a PCC code for any patient between January 2023 and January 2024. Only physicians who had assigned an OHIP code for PCC were included. Approximately 5% of primary care physicians who treat 2% of the population of Ontario are paid entirely by salary and do not bill OHIP. This means that these physicians would not use the PCC diagnostic code even if they were treating a patient for PCC. All other physicians in the province would be required to bill OHIP [21].

### Baseline patient characteristics

We collected patient demographics, which included age, sex, geographic location within the province, rurality, and neighborhood income quintile, immigrant status (provided by Immigration Refugee and Citizenship Canada which includes immigration records since 1985), comorbidities (asthma, chronic obstructive pulmonary disease [COPD], hypertension, diabetes, and congestive heart failure), and marginalization as defined by the Ontario Marginalization Index (ON-Marg). Comorbidities were identified using algorithms validated against a clinical reference standard developed at ICES which make use of a combination of OHIP diagnostic and billing codes and ICD-10 codes from hospitalizations and ED visits. The ON-Marg is a geographically-defined variable that includes four dimensions: households and dwellings (measures the types and density of residential accommodations), material resources (assesses ability to obtain and access basic material needs), age and labour force (includes information describing the number of seniors and individuals not participating in the labour force), and racialized and newcomer population (describes the number of recent immigrants and self-identified visual minorities living in a community). Characteristics of the rest of the population of Ontario were also determined, as a comparison for the PCC cohort [22, 23].

### Outcomes

#### Patient outcomes

Total healthcare utilization from January 2017 – January 2024 was captured for all patients in the patient cohort, including outpatient visits, emergency department visits, and days spent in hospital. Each individual visit and day in hospital was considered a separate healthcare encounter and summed into a total score. For example, 1 outpatient visit, 1 ED visit, and 3 days in hospital would be considered as a total of 5 healthcare encounters. Healthcare utilization was further broken down by reason for the encounter (i.e. respiratory disease, circulatory disease). Healthcare use was examined during three different time periods: pre-pandemic (January 1st 2017 – March 31st 2020), pre-PCC (April 1st 2020–24 weeks pre-index date), and post-PCC (24 weeks pre-index date – January 1st 2024). Healthcare use was captured pre-pandemic and during the pandemic to establish two different baseline levels of use as it was considered likely that pandemic restrictions would affect healthcare utilization patterns. The 24-week pre-index date time period was used as it was considered likely that healthcare use during this time period could be related to PCC. The 24-week time period was established based on a combination of clinical considerations and the PCC disease definition. Technically a minimum of 3 months from the date of infection would be required for a patient to meet the criteria for a PCC diagnosis as patients are required to be experiencing symptoms 3 months following acute infection and to continue experiencing symptoms for at least 2 months which could extend the time period beyond 3 months if new symptoms arose. Additionally, PCC is a diagnosis of exclusion requiring other illnesses to be ruled out which could extend the time period required for diagnosis. Considering these factors, 24 weeks (approximately 6 months) was thought to be a likely period during which healthcare use could be related to PCC. Differences in healthcare use post-PCC were compared to pre-pandemic and pre-PCC periods.

#### Physician outcomes

The specialty of the physician issuing the code was also captured. Physician specialties come from the Ontario Ministry of Health’s Corporate Provider Database which includes the specialty for which the physician is registered with the province. All potential physician specialties included in this analysis are listed in the Supplementary material (Appendix B).

#### Statistical analysis

Crude monthly incidence rates of patients receiving a PCC code for the first time within the entire population of Ontario were calculated.

Patient characteristics were determined for the entire patient cohort and stratified by age (0–18,19–30,31–40,41–65, and > 65 years). Categorical data were reported as counts and percentages and continuous data were reported as medians and interquartile ranges (IQRs). Standardized mean differences (SMDs) were used to identify potentially meaningful distributional imbalances between age groups. An SMD of > 0.1 was considered to represent a potentially meaningful difference.

Incidence rates per month was plotted for patients receiving their first code.

Patient healthcare use was calculated as a per person-year rate. Differences in mean healthcare use from pre-pandemic to post-index date and pre-index date to post-index date were calculated to establish and compare healthcare utilization post-PCC to baseline measures. Confidence intervals (CIs) were calculated using 1000 bootstrap replicates. Additionally, healthcare use in the 24 weeks (approximately six months) both leading up to and following the receipt of the PCC code was plotted.

To investigate how the PCC population compares to the population of Ontario, PCC code received vs. not received was captured in the entire population of Ontario. Univariable logistic regression was used to calculate odds ratios, confidence intervals, and p-values for sex and age groups in PCC compared to the rest of the population of Ontario. Additional individual logistic regression models controlling for age and sex were also created for comorbidities (asthma, COPD, hypertension, diabetes, and congestive heart failure), geographic location in the province, rurality, immigrant status, and marginalization across all four dimensions (households and dwellings, material resources, age and labour force, and racialized and newcomer populations).

Missing data were handled by including a missing data category for categorical data.

## Results

### Patient cohort

A total of 7,343 individuals received at least one code for PCC during the study period. The median age of these individuals was 62 years and 60.3% were female. Individuals received a median of one (interquartile range 1–1) PCC code during the observation period and had a median of two positive PCR COVID-19 tests and three COVID-19 vaccinations before they received the PCC code. Complete descriptive statistics, overall and stratified by age group are included in Table 1.

**Table 1:** Patient characteristics overall and stratified by age. Continuous variables are reported as medians and interquartile ranges and categorical variables are reported as counts and percentages. Standardized mean differences (SMD) were used to identify distributional imbalances between age groups with an SMD of >0.1 considered to be potentially meaningful

		Age					
Variables	Overall	0–18	19–30	31–40	41–65	> 65	SMD
N	7343	249	477	714	2641	3262	
Sex (Female) (%)	4427 (60.3)	124 (49.8)	307 (74.4)	484 (77.8)	1747 (66.1)	1765 (54.1)	0.193
Age (Median [IQR])	62.00 [45, 77]	12.00 [3, 16]	26 [23, 29]	36 [33, 38]	54 [48, 60]	79 [73, 86]	
Immigrant (%)	918 (12.5)	0–6 (0.0–5.0)	41 (8.6)	115 (16.1)	497 (18.8)	263 (8.1)	0.276
Geographic Location (%)							0.139
Central (Urban)	2699 (36.8)	84 (33.7)	197 (41.3)	295 (41.3)	1037 (39.3)	1086 (33.3)	
North (Remote Rural)	581 (7.9)	20 (8.0)	35 (7.3)	55 (7.7)	184 (7.0)	287 (8.8)	
South (Rural Urban)	2007 (27.3)	86 (34.5)	126 (26.4)	180 (25.2)	700 (26.5)	915 (28.1)	
West (Rural Urban)	2056 (28.0)	59 (23.7)	119 (24.9)	184 (25.8)	720 (27.3)	974 (29.9)	
Income Quintile and Rurality (%)							0.222
1 (Lowest)	1227 (16.7)	23 (9.2)	85 (17.8)	127 (17.8)	381 (14.4)	611 (18.7)	
2	1310 (17.8)	36 (14.5)	98 (20.5)	133 (18.6)	451 (17.1)	592 (18.1)	
3	1273 (17.3)	53 (21.3)	101 (21.2)	138 (19.3)	460 (17.4)	521 (16.0)	
4	1332 (18.1)	49 (19.7)	79 (16.6)	136 (19.0)	523 (19.8)	545 (16.7)	
5 (Highest)	1427 (19.4)	55 (22.1)	76 (15.9)	117 (16.4)	563 (21.3)	616 (18.9)	
Rural (Yes) (%)	758 (10.3)	33 (13.3)	37 (7.8)	61 (8.5)	258 (9.8)	369 (11.3)	
Missing	16 (0.2)	0 (0.0)	1 (0.2)	2 (0.3)	5 (0.2)	8 (0.2)	
Comorbidities (%)							
Hypertension	3441 (46.9)	0–6 (0.0–5.0)	11 (2.3)	53 (7.4)	832 (31.5)	2545 (78.0)	1.149
Diabetes	1623 (22.1)	0–6 (0.0–5.0)	7 (1.5)	29 (4.1)	417 (15.8)	1170 (35.9)	0.554
COPD	1396 (19.0)	0–6 (0.0–5.0)	0–6 (0.0–5.0)	0–6 (0.0–5.0)	363 (13.7)	1031 (31.6)	0.513
Asthma	1705 (23.2)	50 (20.1)	126 (26.4)	196 (27.5)	656 (24.8)	677 (20.8)	0.096
Congestive Heart Failure	725 (9.9)	0–6 (0.0–5.0)	0–6 (0.0–5.0)	0–6 (0.0–5.0)	75 (2.8)	649 (19.9)	0.348
Positive COVID- 19 PCR Test Pre PCC (%)	2 [0, 4]	1 [0, 2]	1 [0, 3]	2 [0, 4]	2 [0, 4]	2 [1, 4]	0.207
Vaccination Pre PCC Diagnosis (Median [IQR])	3 [3, 5]	2 [0, 2]	3 [2, 3]	3 [2, 4]	3 [2, 4]	4 [3, 5]	0.859
Total PCC Codes (Median [IQR])	1 [1, 1]	1 [1, 1]	1 [1, 1]	1 [1, 1]	1 [1, 1]	1 [1, 2]	0.182
Post PCC Code, days (Median [IQR])	221 [110, 318]	265 [141, 336]	252 [138, 333]	246 [134, 335]	258 [134, 329]	151 [89, 295]	0.207
Pre PCC Code, days (Median [IQR])	1162 [1077, 1279]	1132 [1063, 1258]	1148 [1067, 1262]	1153 [1065, 1265.75]	1140 [1070, 1265]	1206 [1089, 1293]	0.132
Pre Pandemic, days (Median [IQR])	1186 [1186, 1186]	1186 [1186, 1186]	1186 [1186, 1186]	1186 [1186, 1186]	1186 [1186, 1186]	1186 [1186, 1186]	< 0.01
Ontario Marginalization Index (%)							
House and Dwellings							0.281
1 (Lowest Marginalization)	1289 (17.6)	52 (20.9)	85 (17.8)	116 (16.2)	546 (20.7)	490 (15.0)	
2	1327 (18.1)	59 (23.7)	85 (17.8)	138 (19.3)	504 (19.1)	541 (16.6)	
3	1331 (18.1)	66 (26.5)	76 (15.9)	124 (17.4)	485 (18.4)	580 (17.8)	
4	1423 (19.4)	42 (16.9)	88 (18.4)	114 (16.0)	514 (19.5)	665 (20.4)	
5 (Highest Marginalization)	1923 (26.2)	28 (11.2)	140 (29.4)	219 (30.7)	573 (21.7)	963 (29.5)	
Material Resources							0.118
1 (Lowest Marginalization)	1624 (22.1)	63 (25.3)	112 (23.5)	168 (23.5)	595 (22.5)	686 (21.0)	
2	1658 (22.6)	56 (22.5)	104 (21.8)	164 (23.0)	633 (24.0)	701 (21.5)	
3	1484 (20.2)	59 (23.7)	90 (18.9)	134 (18.8)	547 (20.7)	654 (20.0)	
4	1236 (16.8)	33 (13.3)	82 (17.2)	115 (16.1)	437 (16.5)	569 (17.4)	
5 (Highest Marginalization)	1291 (17.6)	36 (14.5)	86 (18.0)	130 (18.2)	410 (15.5)	629 (19.3)	
Age and Labour Force							0.345
1 (Lowest Marginalization)	1555 (21.2)	76 (30.5)	157 (32.9)	227 (31.8)	682 (25.8)	413 (12.7)	
2	1316 (17.9)	48 (19.3)	103 (21.6)	152 (21.3)	534 (20.2)	479 (14.7)	
3	1257 (17.1)	45 (18.1)	79 (16.6)	123 (17.2)	493 (18.7)	517 (15.8)	
4	1277 (17.4)	33 (13.3)	68 (14.3)	123 (17.2)	436 (16.5)	617 (18.9)	
5 (Highest Marginalization)	1888 (25.7)	45 (18.1)	67 (14.0)	86 (12.0)	477 (18.1)	1213 (37.2)	
Racialized and Newcomer Populations							0.168
1 (Lowest Marginalization)	1160 (15.8)	33 (13.3)	62 (13.0)	93 (13.0)	373 (14.1)	599 (18.4)	
2	1468 (20.0)	45 (18.1)	75 (15.7)	131 (18.3)	503 (19.0)	714 (21.9)	
3	1502 (20.5)	45 (18.1)	88 (18.4)	134 (18.8)	556 (21.1)	679 (20.8)	
4	1587 (21.6)	60 (24.1)	106 (22.2)	176 (24.6)	572 (21.7)	673 (20.6)	
5 (Highest Marginalization)	1576 (21.5)	64 (25.7)	143 (30.0)	177 (24.8)	618 (23.4)	574 (17.6)	
Missing	50 (0.7)	2 (0.7)	3 (0.6)	3 (0.4)	19 (0.7)	23 (0.7)	
Death (%)	488 (6.6)	0–6 (0.0–5.0)	0–6 (0.0–5.0)	0–6 (0.0–5.0)	34 (1.3)	452 (13.9)	0.317

*Continuous Variable; *IQR* interquartile range, *SMD* Standardized Mean Difference, *ED* Emergency Department, *SDS* Same Day Surgery, *CIHI* Canadian Institute for Health Information, *COPD* Chronic Obstructive Pulmonary Disease, *PCR* Polymerase Chain Reaction, *PCC* Post-COVID- 19 Condition, *SD* standard deviation, *IQU* interquartile range

**Fig. 1:**
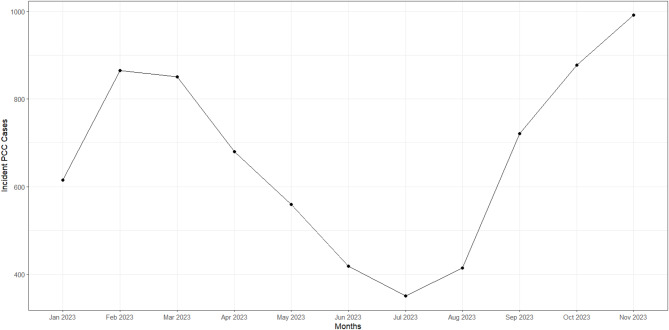
Incidence rates of patients receiving their first PCC code by month

Incidence of first codes were recorded between January and November 2023, no new PCC codes were recorded in December 2023. Peaks in incidence rates were observed in February and November 2023 (Fig. 1).

### PCC compared to the entire population

When compared to the entire population of Ontario, individuals with PCC were more likely to be older, female, from northern Ontario (a remote and largely rural population with less access to healthcare), and have comorbidities. When examining ON-Marg dimensions individuals, living in neighbourhoods with the greatest racialized and newcomer population and higher levels of material deprivation were less likely to have a PCC code. All odds ratios are included in Table 2.

**Table 2:** Univariable and sex and age controlled logistic regression models using post-COVID-19 condition (PCC) status as the dependent variable for the entire population of Ontario, Canada demonstrating how the PCC population differs from the non-PCC population of Ontario

Variable	Odds Ratio (CI)	*P*-Value
Sex and Age		
Sex (Female)	1.392 (1.33-1.46)	< 0.001
Age (Reference 0–18)		
19–30	2.426 (2.081-2.827)	< 0.001
31–40	4.768 (4.173-5.449)	< 0.001
41–65	6.777 (5.934-7.741)	< 0.001
> 65	14.578 (12.815-16.584)	< 0.001
Sex and Age Adjusted Variables		
Geographic Location in the Province (Reference Central Ontario)		
Northern Ontario	1.326 (1.212-1.451)	< 0.001
Southern Ontario	0.949 (0.896-1.006)	0.0767
Western Ontario	0.95 (0.897-1.006)	0.0791
Immigrant	0.601 (0.56-0.644)	< 0.001
Rural (Reference No)		
Yes	0.933 (0.865-1.006)	0.07
Missing	0.924 (0.557-1.534)	0.7599
Comorbidities		
Asthma	1.808 (1.713-1.909)	< 0.001
Congestive Heart Failure	2.643 (2.439-2.865)	< 0.001
Chronic Obstructive Pulmonary Disease	1.931 (1.815-2.054)	< 0.001
Hypertension	1.557 (1.472-1.647)	< 0.001
Diabetes	1.306 (1.232-1.384)	< 0.001
Sex and Age Adjusted Ontario Marginalization Index Dimensions		
Material Resources (Reference 1 Lowest Marginalization)		
2	0.965 (0.901-1.033)	0.3026
3	0.921 (0.858-0.988)	0.022
4	0.855 (0.794-0.921)	< 0.001
5 (Highest Marginalization)	0.894 (0.831-0.961)	0.0026
Missing	0.857 (0.647-1.136)	0.284
Racialized and Newcomer Population (Reference 1 Lowest Marginalization)		
2	1.194 (1.105-1.289)	< 0.001
3	1.21 (1.121-1.306)	< 0.001
4	1.115 (1.034-1.203)	0.0048
5 (Highest Marginalization)	0.873 (0.809-0.943)	< 0.001
Missing	0.98 (0.738-1.301)	0.8878
Age and Labour Force (Reference 1 Lowest Marginalization)		
2	1.078 (1.002-1.161)	0.0449
3	1.112 (1.032-1.199)	0.0053
4	1.107 (1.027-1.193)	0.0076
5 (Highest Marginalization)	1.292 (1.206-1.384)	< 0.001
Missing	1.03 (0.777-1.365)	0.8378
Households and Dwellings (Reference 1 Lowest Marginalization)		
2	1.136 (1.052-1.227)	0.0011
3	1.13 (1.047-1.22)	0.0018
4	1.188 (1.102-1.281)	< 0.001
5 (Highest Marginalization)	1.31 (1.22-1.406)	< 0.001
Missing	1.067 (0.804-1.415)	0.6535

*CI* confidence interval

**Fig. 2:**
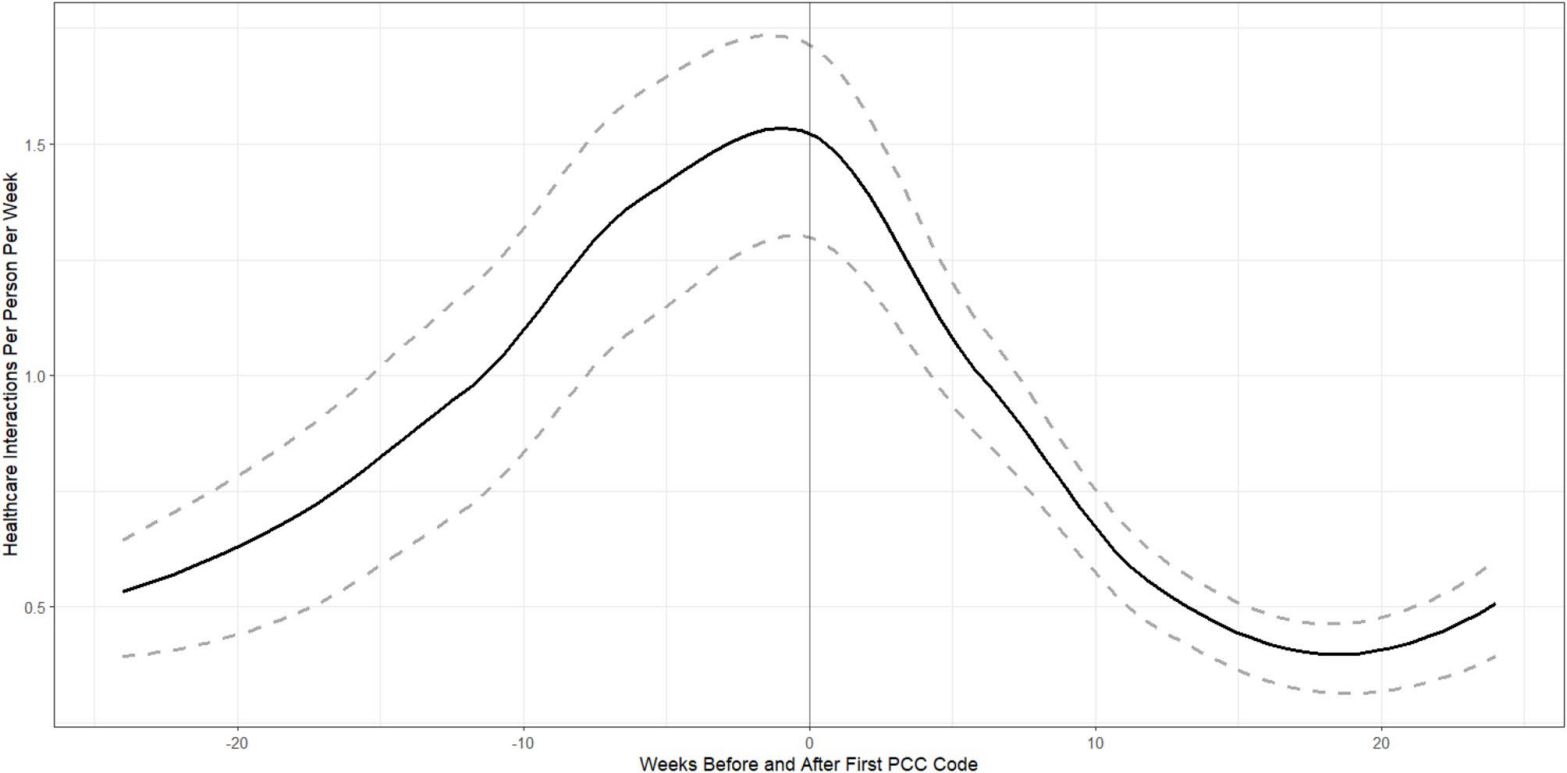
Smoothed weekly mean healthcare utilization rate with confidence intervals (dashed grey lines) 24 weeks before and after receiving a PCC code (vertical line at 0)

Individuals were found to have substantially higher per person-year rates of healthcare use following the receipt of a PCC code (rate = 100.61, 95% CI: 93.39–107.73) when compared to both pre-pandemic (rate = 14.59, 95% CI: 13.63–15.61) and pre-PCC (rate = 27.43, 95% CI: 25.01–30.27) periods. When investigating the plot of healthcare utilization, a substantial rise was observed in the 24 weeks leading up to and following the receipt of a PCC code (Fig. 2).

When stratified by disease type, post-PCC healthcare use was elevated across all disease types. However, it was highest for the respiratory (rate = 14.0 CI: 12.1–15.9), circulatory (rate = 12.2 CI: 10.5–14.2), symptoms signs and abnormal clinical and laboratory findings not classified elsewhere (rate = 8.2 CI: 7.2–9.6), infectious (rate = 7.3 CI:6.4–8.2), and mental and behavioural (rate = 7.8 CI: 7.0–8.7) categories. Healthcare use belonging to categories not listed was also elevated (rate = 35.6 CI: 31.9–39.4). Healthcare use stratified by healthcare use type is included in Table 3.

**Table 3:** Healthcare utilization rates per person per year pre-pandemic (January 1st 2017 – March 31st 2020), pre-PCC from the start of the pandemic to 24 weeks pre-index date (April 1st 2020–24 weeks pre-index date), and post-PCC from 24 weeks pre-index date to the study end date (24 weeks pre-index date – January 1st 2024). Rates post-PCC and pre-PCC and post-PCC and pre-pandemic were calculated. Confidence intervals were calculated using 1000 bootstrap replicates

	Time Period			Rate Difference	
Healthcare Use Category	Post-PCC Rate Mean (CI)	Pre-PCC Rate Mean (CI)	Pre-Pandemic Rate Mean (CI)	Post-PCC and Pre-PCC (CI)	Post-PCC and Pre-Pandemic (CI)
HCU By Care Type					
Days in Hospital (DAD)	41.9 (36. 6–48.3)	13.3 (11.1–15.9)	3.9 (4.0–4.8)	28.5 (22.9–34.3)	38.0 (32.5–43.8)
ED Visits (NACRS)	3.1 (2.9–3.2)	0.8 (0.8–0.8)	0.7 (0.6–0.7)	2.3 (2.1–2.4)	2.4 (2.3–2.5)
Outpatient Visits (OHIP)	55.6 (53.7–57.7)	13.3 (12.9–13.6)	10.1 (9.8–10.3)	42.3 (40.5–44.4)	45.6 (43.5–47.6)
HCU by Disease/Disorder Type					
Diseases of the Respiratory System	14.0 (12.1–15.9)	3.0 (2.3–3.7)	1.6 (1.4–1.9)	11.0 (9.1–12.9)	12.3 (10.5–14.4)
Diseases of the Circulatory System	12.2 (10.4–14.2)	3.5 (3.0–4.1)	1.6 (1.5–1.8)	8.7 (6.9–10.8)	10.6 (8.8–12.7)
Diseases of the Digestive System	5.3 (4.4–6.4)	1.7 (1.5–2.0)	1.19 (0.9–1.6)	3.6 (2.7–4.6)	4.126 (3.1–5.3)
Infectious and Parasitic Diseases	7.3 (6.4–8.2)	1.3 (1.1–1.5)	0.5 (0.4–0.6)	6.0 (5.2–7.0)	6.82 (6.0–7.7)
Mental, Behavioural and Neurodevelopmental Disorders	7.8 (7.0–8.7)	2.3 (2.0–2.6)	1.3 (1.2–1.5)	5.5 (4.7–6.4)	6.4 (5.6–7.4)
Diseases of the Musculoskeletal System and Connective Tissue	3.2 (2.7–3.7)	1.5 (1.3–1.9)	1.1 (1.0–1.2)	1.7 (1.1–2.3)	2.1 (1.6–2.6)
Diseases of the Nervous System	1.9 (1.5–2.4)	0.6 (0.5–0.8)	0.2 (0.2–0.3)	1.3 (0.9–1.9)	1.702 (1.3–2.2)
Endocrine, Nutritional and Metabolic Disease	3.5 (2.8–4.4)	1.3 (1.1–1.6)	0.8 (0.7–0.9)	2.2 (1.5–3.2)	2.7 (2.0–3.6)
Diseases of the Blood and Blood-Forming Organs	1.6 (1.1–2.1)	0.4 (0.3–0.5)	0.2 (0.1–0.2)	1.2 (0.7–1.7)	1.373 (0.9–2.0)
Symptoms Signs and Abnormal Clinical and Laboratory Findings Not Classified Elsewhere	8.2 (7.2–9.5)	2.3 (2.0–2.6)	1.3 (1.1–1.4)	6.0 (4.8–7.2)	7.0 (6.0–8.2)
All other healthcare use categories	35.6 (31.9–39.4)	9.6 (8.4–11.0)	4.7 (4.3–5.3)	26.1 (22.5–30.1)	30.9 (27.5–34.7)
Total					
Total	100.6 (93.4–107.7)	27.4 (25.0–30.3)	14.6 (13.6–15.6)	73.2 (66.4–80.0)	86.0 (79.1–92.7)

*PCC* post-COVID- 19 condition, *CI* confidence interval, *DAD* Discharge Abstract Database, *NACRS* National Ambulatory Care Reporting System, *OHIP* Ontario Health Insurance Plan

No continuous data were missing, categorical missing data are included in Table 1.

### Physicians

The most common physician specialties to issue PCC codes were family medicine (*n* = 675) and internal medicine (*n* = 157). There were nine other specialties in which at least six physicians issued PCC codes. These included respirologists, pediatricians, geriatricians, critical care physicians, medical oncologists, haematologists, physical medicine physicians, infectious disease physicians, and anaesthesiologists. The number of physicians issuing codes and the total number of codes issued stratified by specialty is included in Table 4.

**Table 4:** Post COVID-19 condition codes issued stratified by physician specialty

Specialty Name	Number of Physicians	Total Codes Issued	Mean number of Codes Per Physician (SD)
General Practitioner	675	8549	12.7 (25.9)
Internal Medicine	157	2917	18.6 (30.3)
Respiratory Disease	21	177	8.4 (10.5)
Paediatrics	20	40	2 (1.0)
Geriatrics	11	128	11.6 (14.5)
Critical Care Medicine	11	61	5.5 (4.9)
Medical Oncology	10	142	14.2 (16.5)
Haematology	10	63	6.3 (5.9)
Physical Medicine	9	349	38.8 (88.0)
Infectious Disease	8	110	13.8 (13.7)
Anaesthesia	6	34	5.7 (4.7)

*SD* Standard Deviation

## Discussion

We conducted a retrospective cohort study in Ontario, Canada that investigated the characteristics and healthcare utilization rates of people who received a PCC diagnostic code and the physicians who issued the code. Our study found a smaller number of people received a PCC code than expected based on, Health Canada’s estimate of PCC. When investigating how the PCC population compares to the general population of Ontario [5, 24] higher use of PCC diagnostic codes were observed in females, older individuals, and patients with comorbidities. Patients who received a PCC code were also found to have substantially higher rates of healthcare use leading up to and following a PCC diagnosis compared to their pre-pandemic healthcare use. From the physician perspective, family physicians and internal medicine specialists used the greatest number of PCC diagnostic codes.

The factors associated with use of a PCC code are consistent with what has previously been observed in the literature [5, 24]. However, racialized and newcomer populations and those of lower socioeconomic status were disproportionately affected by COVID-19 in Canada [25, 26]. However, our study found that neighbourhoods with the greatest proportion of racialized and newcomer population and greater levels of material deprivation were underrepresented in the PCC data. Pfaff et al. found similar trends in an American PCC population when using ICD-10 codes to identify patients [27]. These findings could suggest that people in these neighbourhoods are not as likely to be affected by PCC. This could be possible as immigrants in Canada have been observed to be generally healthier and to use less healthcare than their non-immigrant counterparts [28]. Additionally, in Canada race has not been found to significantly affect the likelihood of an individual having a family physician, suggesting that access to care may not be driving this disparity [29]. However, it is also possible that individuals in these populations experience barriers to care that are preventing them from being treated for or receiving a PCC diagnosis. This will need to be explored with more granular individual data as only geographically defined variables were used in this study.

Consistent with the previous literature, our study found that a PCC diagnosis is associated with a substantial increase in healthcare utilization [4, 15]. This increase is particularly centered around the PCC diagnosis date with a dramatic increase leading up to the diagnosis followed by a substantial decrease (Fig. 2). This is consistent with healthcare utilization patterns observed when diagnosing a chronic disease [30–32]. Although, it is unclear why the drop off in healthcare utilization occurs, a possible explanation is that once a diagnosis is received, patients no longer use healthcare for diagnostic reasons and physicians and patients can develop a plan to manage PCC symptoms more independently, thereby reducing subsequent healthcare utilization. Given the relatively short follow up time for this study, it is not clear if this decrease is permanent, or if healthcare utilization will increase again following this period.

In the 24-weeks leading up to and following receipt of the PCC code, increases in all types of healthcare use were observed with the greatest increases being observed in respiratory disease, circulatory disease, infectious diseases, mental and behavioural disorders, and symptoms signs and abnormal clinical and laboratory findings not classified elsewhere [6]. This roughly aligns with the cluster of symptoms most commonly attributed to PCC, with the exception of the musculoskeletal category [6]. The lack of increase in musculoskeletal healthcare use could be because these issues are being classified as irregular signs and symptoms, for example, fatigue, a lack of coordination, or unsteadiness on one’s feet. The greatest observed increase in healthcare use was healthcare from all other causes not listed in Table 3. This reinforces that healthcare usage is increasing for a very wide array of ailments in this population, which may be consistent with the multi-system nature of PCC and the difficulty associated with diagnosing and treating this condition. Surprisingly, neurological healthcare use was not dramatically elevated [6]. This may indicate that neurological conditions were less prevalent or severe than other symptoms. However, it is also possible that this is a product of low use of the PCC diagnostic code by neurologists or barriers to care that prevent patients from visiting a neurologist. Given that some of the most frequently described symptoms of PCC are neurological, it would be expected that these specialties would treat patients with PCC [6]. Additional outreach or education may be needed to ensure that the PCC code is being used across all specialties.

There is currently a lack of an objective gold standard or recognized biomarker for diagnosing PCC and establishing its incidence and prevalence. However, given its infrequent use we believe that it is unlikely that this code is capturing the majority of PCC cases in Ontario. We determined that in 2023, 7,343 individuals were diagnosed with PCC, which is significantly less than our rough estimate of 13,000–26,000 expected cases [11]. Along with the limited use of the code by certain physician specialities, we think that use of the diagnostic code to monitor PCC will underestimate the true burden of the disease in Ontario. It is possible that this code could act as an indicator of increasing or decreasing PCC in the province, even though absolute numbers may not be accurate. However, much more work would be required to investigate the code, particularly the prevalence of use among physicians as this will affect the number of codes issued. The characteristics of the patients who have been collected in this sample appear to be consistent with what has been observed in the literature when considering both demographic factors and healthcare utilization patterns. This suggests that the patients in this cohort likely do have PCC, although all PCC phenotypes may not have been captured. The observed increase in healthcare utilization rates also suggests that the PCC code could be predominantly capturing severe cases of PCC. Given the characteristics of the identified PCC population, we feel that, with judicious application, the diagnostic code can be a useful tool to study a large population of affected individuals.

This study had a number of limitations. First, without symptom data, our findings are subject to misclassification, as we are unable to confirm that people who received a PCC code met the PCC disease definition. However, when considering the characteristics of the population, the history of positive COVID-19 tests in most patients, and the increase in healthcare use, it is likely that a majority of patients who received a PCC code had PCC. Second, the results of this study may not generalize to populations outside of Ontario where demographics and the healthcare system differ. Third, publicly available testing was ended on November 21st, 2021. This means that patients may have contracted COVID-19 for which they did not receive a PCR test, as home testing was predominantly used in Ontario during this period. This means that the number of COVID-19 infections may have been underestimated as only COVID-19 infections confirmed by PCR testing were included. Fourth, not all physicians in Ontario would be required to issue billings to OHIP as physicians who work in community health centers (CHC) are paid by salary and do not bill OHIP. This would mean that CHC physicians would not bill for PCC and may contribute to the underestimation of PCC in Ontario. However, these physicians only account for approximately 5% of primary care physicians and have been found to treat approximately 2% of the population of Ontario indicating that this was not likely to result in a large effect on PCC code use [21]. Lastly, this code was only introduced in January 2023 we only collected data until January 2024 and no new cases were observed in December 2023, a longer time horizon is likely necessary to allow for more physicians to become familiar with and learn about the code. Additionally, earlier strains and individuals who were infected prior to the vaccines would not be captured.

## Conclusion

It appears that this newly developed PCC code can not be used to accurately monitor the burden of PCC in Ontario. However, it may be useful as an indicator of trends in PCC diagnostic code use over time. In addition, the PCC code, is an efficient way to capture a large, diverse number of probable PCC patients who appear to be representative of the PCC population based on the limited literature available and thus could be used to investigate this population. In order to more rigorously validate this code, gold standard diagnostic criteria or a biomarker will need to be identified as presently PCC appears to be a somewhat subjective diagnosis. The diagnostic code was predominantly issued by family and internal medicine physicians, suggesting a need to improve implementation and use across all specialties. Our findings provide new insights about healthcare utilization patterns of PCC patients as well as an indication that there is value in further exploring this PCC code. It may be necessary for physicians to receive education on the PCC code so that it can be used more reliably.

## Supplementary Information

Supplementary Material 1.

## Data Availability

The dataset from this study is held securely in coded form at ICES. While legal data sharing agreements between ICES and data providers (e.g., healthcare organizations and government) prohibit ICES from making the dataset publicly available, access may be granted to those who meet pre-specified criteria for confidential access, available at www.ices.on.ca/DAS (email: das@ices.on.ca). The full dataset creation plan and underlying analytic code are available from the authors upon request, understanding that the computer programs may rely upon coding templates or macros that are unique to ICES and are therefore either inaccessible or may require modification.

## Abbreviations

OHIP: Ontario Health Insurance Plan
PCC: Post-COVID-19 condition
OR: Odds ratio
CI: Confidence interval
PCR: Polymerase chain reaction
PHIPA: Personal Health Information Protection Act
ICD- 10: International Classification of Diseases Tenth Revision
ED: Emergency department
SDS: Same day surgery
COPD: Chronic obstructive pulmonary disease
ON-Marg: Ontario Marginalization Index
IQR: Interquartile ranges
SMD: Standardized mean differences
