# Supplementary material for: Investigating use of diagnostic codes for post-COVID- 19 condition in Ontario health administrative data
Source: BMC Health Serv Res. 2025 May 14;25:694. doi: 10.1186/s12913-025-12751-4 (PMC12077028; doi:10.1186/s12913-025-12751-4)
Supplement: Supplementary file 1 — Supplementary Material 1. [file 12913_2025_12751_MOESM1_ESM.docx]

Supplementary Material

Appendix A: Description of databases used to create dataset

| Database Name | Description |
| --- | --- |
| Assistive Device Program (ADP) | The assistive device program contains information on individuals in Ontario who have a disability that requires the use of an assistive device (for example a wheelchair hearing aid, or long-term oxygen) for six months or longer |
| ASTHMA | The Ontario Asthma Cohort (sensitive) and Ontario Asthma Specific Cohort contain all Ontario asthma patients identified since 1991 |
| Client Agency Program Enrollment (CAPE) | This database includes information on all patients registered with a primary care organization in Ontario. This includes information on patients' association with an individual physician and primary care organization. |
| Continuing Care Reporting System (CCRS) | Contains demographic, clinical, functional, and resource utilization information on individuals receiving continuing care in hospital or long-term care facilities in Canada. |
| CENSUS | Includes information from the 2021 Canadian Census of Population. This includes geographic information on provinces, census metropolitan areas, communities, and census tracts |
| Congestive Heart Failure (CHF) | Is a database that includes all individuals in Ontario with identified with congestive heart failure in Ontario since 1991 |
| Immigration, Refugees and Citizenship Canada Permanent Resident Database (CIC) | This database includes immigration application records on individuals who applied to land in Ontario. This includes permanent residents of Canada's demographic information. |
| CONTACT | This database tracks individual's contact with the healthcare system |
| Chronic Obstructive Pulmonary Disease (COPD) | This database includes all individuals with COPD identified in Ontario since 1991 |
| Corporate Providers Database (CPDB) | Provides information on groups/healthcare providers who are eligible to receive payments from the Ontario Health Insurance Plan |
| Canadian Institute for Health Information (CIHI) Discharge Abstract Database (DAD) | This database contains information on all hospital discharges within the province of Ontario |
| Home Care Database (HCD) | This database contains information on all home care services provided or organized by Local Health Integration Networks. |
| HYPER | This dataset includes information on all individuals with hypertension in Ontario identified since 1991 |
| Institution Information System (INST) | This dataset contains information on health care institutions in Ontario funded by the ministry of health and long-term care |
| ICES Physician Data Base (IPDB) | This database contains information about all physicians in Ontario |
| Local Health Integration Network (LHIN) | This database contains information on 14 different geographic locations within Ontario |
| National Ambulatory Care Reporting System (NACRS) | This database contains information on all hospital-based ambulatory and community-based care. This includes information on emergency department visits |
| Ontario Cancer Registry (OCR) | This is a provincial database that contains information on all Ontario residents diagnosed with cancer |
| Ontario Diabetes Database (ODD) | This database contains a list of all individuals in Ontario with any type of nongestational diabetes identified since 1991. Type1 and type 2 are not distinguished between. |
| Ontario Health Insurance Plan (OHIP) | This database includes all claims paid for by the Ontario Health Insurance Plan (OHIP). This covers all healthcare providers who submit claims to OHIP. OHIP covers almost all residents of Ontario. |
| Ontario Laboratories Information System (OLIS) | This database includes information for lab tests ordered in Ontario. Some tests include results but not all of them |
| Ontario Mental Health Reporting System (OMHRS) | This database collects data on adults who are designated as inpatients for a mental health reason at general healthcare facilities, psychiatric facilities, and specialty psychiatric facilities |
| Ontario Marginalization Index (ON-MARG) | This a database that includes information on the four major dimensions that are thought to comprise the concept of marginalization. These include households and dwellings, material resources, age and labour force, and racialized and newcomer populations |
| Ontario Rheumatoid Arthritis Database (ORAD) | This is a database that includes all individuals in Ontario with rheumatoid arthritis identified since 1991. |
| Office of the Registrar General - Deaths (ORGD) | This is a dataset that contains information on all deaths and causes of death registered in Ontario since 1990. |
| Ontario Population Estimates and Projections (POP) | This dataset contains estimates of the Ontario population by sex, age, and geographic location |
| Registered Persons Database (RPDB) | This database provides basic demographic information on anyone in Ontario who has ever received a health card number. |
| CIHI Same Day Surgery Database (SDS) | The Same Day Surgery database contains administrative, clinical, and demographic information on day surgery procedures |
| COVID-19 Integrated Testing Data (C19INTGR) | This is a comprehensive dataset that includes all available COVID-19 polymerase chain reaction diagnostic laboratory results in Ontario |
| Ontario COVID-19 Vaccine Data (COVAXON) | This dataset includes all COVID-19 vaccinations including dates delivered in Ontario until December 2023 |

Appendix B – All potential physician specialties included in the analysis

Family Practice and Practice In General, Anaesthesia, Dermatology, General Surgery, Neurosurgery, Community Medicine, Orthopaedic Surgery, Geriatrics, Plastic Surgery, Cardiovascular and Thoracic Surgery, Critical Care Medicine, Emergency Medicine, Internal Medicine, Endocrinology, Nephrology, Vascular Surgery, Neurology, Psychiatry, Obstetrics and Gynaecology, Genetics, Ophthalmology, Otolaryngology, Paediatrics, Laboratory Medicine, Physical Medicine, Diagnostic Radiology, Radiation Oncology, Urology, Gastroenterology, Medical Oncology, Palliative Medicine, Infectious Disease, Respiratory Disease, Rheumatology, Dental Surgery, Oral Surgery, Orthodontics, Paedodontics, Periodontics, Oral Pathology, Optometry / Optometrist, Chiropody (Podiatry), Cardiology, Haematology, Clinical Immunology, Nuclear Medicine, and Thoracic Surgery
